# Polymorphisms within the Promoter Region of the Gamma Interferon (IFN-γ) Receptor1 Gene are Associated with the Susceptibility to Chronic HBV Infection in an Iranian Population

**DOI:** 10.5812/hepatmon.7283

**Published:** 2012-11-06

**Authors:** Sayyad Khanizadeh, Mehrdad Ravanshad, Seyed Reza Mohebbi, Hamed Naghoosi, Mohamad Abrahim Tahaei, Seyed Dawood Mousavi Nasab, Sara Romani, Pedram Azimzadeh, Azar Sanati, Mohammad Reza Zali

**Affiliations:** 1 Department of Virology, Faculty of Medical Sciences, Tarbiat Modares University, Tehran, IR Iran; 2 Research Center for Gastroenterology and Liver Disease, Shaheed Beheshti University of Medical Sciences, Tehran, IR Iran

**Keywords:** Hepatitis B Virus, Single Nucleotide Polymorphism, Interferon Gamma Receptor, Polymorphism, Restriction Fragment Length

## Abstract

**Background:**

chronic hepatitis B virus (HBV) infection is a multifactorial disease that can result in serious clinical complications. Host genetic background especially the genes that encode immunologic factors like INF-γ and its receptor (IFN-γ R) are critical in the pathogenesis of infection.

**Objectives:**

The current study aimed to investigate the association between two single nucleotide polymorphisms (SNPs) at positions -611 and -56 within the promoter region of gamma interferon receptor1 gene (IFN-γ R1) and chronic HBV infection.

**Materials and Methods:**

Genomic DNA from peripheral blood samples of 200 chronically HBV infected patients and 200 healthy blood donors, as controls, were collected and genomic DNA was extracted by phenol-chloroform method and DNA analysis genotype identification was performed by PCR-RFLP.

**Results:**

The results indicated that both SNP’s frequency had a significant difference in the patient and control groups. At position -56, TT genotype was associated with patient group and P value was 0.002 and at position -611, GG genotype was further observed in control group and P value was 0.006.

**Conclusions:**

Presence of G allele at position -611 within promoter of IFN-γ R1 gene in the enrolled population for the study was related to lower risk of disease, and presence of T allele at position -56 was also related to susceptibility to chronic HBV infection. Men had higher frequency of chronic HBV infection, which might be the result of high risk behavior.

## Background

Chronic HBV infection is one of the most common viral infections worldwide, with about 400 million chronic carriers living in the world (1). More than 90% of HBV infections in adults are self-limited within six months with or without clinical symptoms (acute infection), whereas 5 to 10 % of infections progress towards chronic infection in which the virus is not cleared from the body (2). Chronic infection can lead to serious clinical consequences such as liver cirrhosis and hepatocellular carcinoma (HCC) (3). The host genetic background can play a critical role in the pathogenesis of disease and single nucleotide polymorphism (SNPs) may be effective on the clinical heterogeneity of chronic infection (4). The immune system’s response to primary HBV infection determines the clinical course of HBV infection. The persistent HBV infection is considered a multifactorial disorder, which various factors such as the host genetic background, viral and environment factors are involved in its pathogenesis (5). IFN-γ is a cytokine that plays a crucial role in defense against the infection of intracellular pathogens (especially virus) and the stimulation of an immune mediated inflammatory response (6). The importance of IFN-γ in the clearance of HBV has been recently demonstrated. During an acute self-limited HBV infection, a high level of IFN-γ is secreted by T lymphocytes, which have significant roles on HBV clearance (7). IFN-γ can inhibit the replication of HBV and directly reduces the viral load (8-19). Studies conducted on laboratory animals especially in the transgenic mice have shown that the expression of cytokines can cause decrease in HBV replication, without lethal effect on hepatocytes (7). Nucleotide variations in the regulatory regions of coding cytokine genes and their receptors can lead to modulation in the cytokine expression, a large number of evidences indicate that SNPs within the binding site of transcription factors may cause decrease in the promoter function of a cytokine/receptor complex and thus can affect the symptoms of a disease (7, 8). The host genetic backgrounds, especially (SNPs) can be a marker of the clinical heterogeneity of HBV infection (17, 19). IFN-γ has a heterodimeric receptor that is made up of two chains (IFN-γ R1 and IFN-γ R2), that each mediates a specific function; IFN-γ R1 is involved in binding to the ligand (IFN-γ) and IFN-γ R2 plays a role in the signaling pathway (6). The binding of homodimeric IFN-γ to IFN-γ R1 causes oligomerization of the two receptor subunits. Each receptor subunit is associated with a distinct Janus kinase (Jak), which is activated by reciprocal transphosphorylation upon oligomerization of IFN-γ R1 and IFN-γ R2. Then, Jak activation causes phosphorylation of IFN-γ R1. A latent transcription factor, Stat1, binds to the phosphorylated IFN-γ R1, undergoes tyrosine phosphorylation, and forms homodimers that translocate to the nucleus and regulate transcription of inducible genes by IFN-γ (14). Previous studies have shown that SNPs in the IFN-γ and its receptor can affect the pathogenesis of chronic HBV infection. Also variations in the regulatory genomic regions such as promoter of IFN-γ R have been related to rigorous hepatic fibrosis and in the outcome of Schistosoma mansoni infection (5, 12). Thus polymorphisms within the promoter region of the IFN-γ R1 can cause modulation of the level of gene expression of IFN-γ R1 on the cell surface, a mechanism by which a cell can alter its sensitivity to IFN-γ. According to above mentioned information, IFN-γ/IFN-γR signaling can be important in the pathogenesis of chronic HBV infection, thus investigations in the context of host genetic background especially immune-genetic factors can be valuable in understanding the pathogenesis of chronic HBV infection. In the current report, the correlation between two SNP markers in the IFN-γ R1 gene and susceptibility to chronic HBV infection in an Iranian population were explored.

## Objectives

The current study aimed to investigate the association between two single nucleotide polymorphisms (SNPs) at positions -611 and -56 within the promoter region of gamma interferon receptor1 gene (IFN-γ R1) and chronic HBV infection.

## Materials and Methods

### 3.1. Study Groups

The study included 200 chronic patients positive for HBsAg, who were from different provinces referring to Taleghani hospital during 2010-2011, with symptoms of impaired liver function tests (transaminases twice the level of normal for at least a 6 month period), detectable IgG anti-HBc and/or sonographic, and clinical results compatible with chronic liver disease. 200 blood donors, who were negative for both anti-HBc and HBsAg, were studied as control subjects (Table 1). All individuals who enrolled for the study were from different regions of Iran , that researchers obtained their ethical approvals. Serum HBsAg, HBeAg, anti-HBs, anti-HBe and anti-HBc were assayed by ELISA kits (DRG International Inc., USA), according to the manufacturer’s instruction. None of patients had a history of intravenous drug abuse or extreme alcohol consumption, and all were negative for anti-HCV and anti-HIV. Patients with other liver diseases (hemochromatosis, Wilson’s disease, α-1 antitrypsin deficiency) or history of receiving anti-viral or immunosuppressive drugs were excluded. The blood samples were obtained from each participant and DNA was extracted from buffy coat using the phenol-chloroform method (18)

**Table 1:** Clinical Data of Control and Patient Groups

	CHP	CHS
**Male/female, No.**	130/70	108/92
**HBsAg**	+	−
**Anti-HBsAg**	−	+
**HBeAg**	+	−
**Anti-HBeAg**	−	−
**Anti-HBcAg**	+	−
**ALT**	55.8 ± 62.2	24.6 ± 10.4

Abbreviations: ALT, alanine aminotransferase; CHP, chronic hepatitis B patient; HBsAg, hepatitis b surface antigen ; HBeAg, hepatitis B - antigen ; HBcAg, hepatitis B core antigen ; HCS, healthy controls.

### 3.2. Detection of IFN-γR1 Gene Polymorphism

The regions of IFN-γR gene containing two single nucleotide polymorphisms (SNPs) at positions -56 and -611 were amplified byPCR. The PCR products for analysis were digested by the Hpy188I restriction enzyme (New England Biolab) for -611 SNP and the AfeI restriction enzyme (New England Biolab). (Table 2) shows the Primers and restriction enzymes used for genotyping. The PCR conditions were optimized for -56 SNP as follows: 95°C for 5 min; 33 cycles of 95°C for 30 s, 59°C for 30 s, and 72°C for 30 s; and a final extension at 72°C for 10 min; and for -611 SNP as follows: 94°C for 5 min; 35 cycles of 95°C for 35 s, 61.7°C for 35 s, and 72°C for 35 s; and a final extension at 72°C for 10 min. The PCR products were run on a 1% agarose gel and stained with ethidium bromide for visualization under UV light. The RFLP products for -56 promoter SNPs were visualized on a 2% agarose gel. The products for -611 promoter SNPs were visualized on 18% polyacrylamide gel electrophoresis. To confirm the RFLP genotyping results, 10% of samples were randomly sequenced via direct sequencing by ABI genetic analyzer 3130xl system.

**Table 2:** Primers and Restriction Enzymes for Genotyping

SNP, position	PCR primer sequence (5′- 3′)	Restriction Enzyme	Allele Phenotype
**-611A/G(p)**	F:CTCTTCATGAGAGGCTGTCT	Hpy188I	A: 260bp G:240 + 20bp
	R:TAACTCTTGGAGTTCACCTGG		
**-56T/C(P)**	F:TGCATGACAAGGGGTAGGAG	AfeI	T: 430bp C:339 + 91bp
	R:CAACCAGGTGAAGTCCAAGAG		

### 3.3. Statistical Methods

The data were analyzed using chi-square test according to the Hardy–Weinberg equilibrium (HWE) and independent sample t test, at P < 0.05 significance level. SPSS software version 16.0 was employed to calculate Odds Ratios (ORs) and 95% confidence intervals (95% CI) by binary logistic regression 

## Results

The polymorphisms of IFN-γ R1 promoter were identified in subjects under study (Figure). They were C to T transition at -56 and G to A at -611 relative to promoter site. There were no significant relationships between age and gender of case and control groups and the status of disease (P = 0.380). Mean age of patients and controls were 45.71 ± 15.37 43.99 ± 16.17respectively. As indicated in (Table 3), irrespective of age, and gender factors, a case-control analysis of the population revealed a significant difference in both of the site polymorphisms.

Logistic regression analyses revealed that genotype frequency of -56 TT was significantly higher in cases than in controls with (P < 0.002, OR = 0.469, 95% CI = 0.292-0.754) and genotype frequency of -611GG was notably higher in controls than in cases with (P < 0.006, OR = 0.164, 95% CI = 0.046-0.580). Therefore a significant association between patient and control groups in two polymorphism positions was observed, so that at -56T/C (SNP), homozygosity for the -56 T variant (TT genotype) was associated with persistent HBV infection in the patients group (P = 0.002), and also at -611G/A (SNP) hemozigosity for the -611G variant (GG genotype) was associated with healthy control group (P = 0.006).

**Figure:**
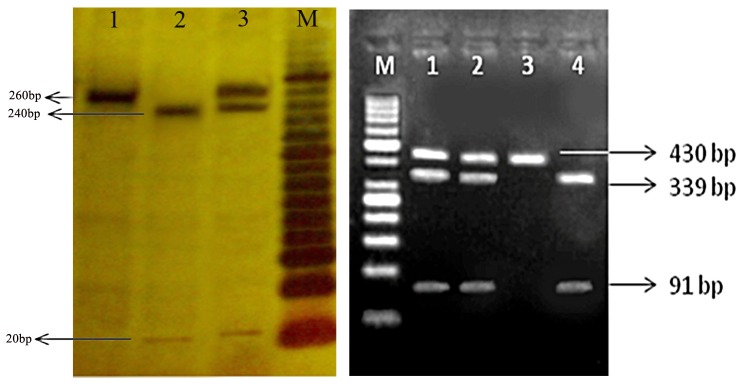
The Results of Digestion by Restriction Enzymes Left) The digestion pattern of Hpy188I restriction enzyme on 18% polyacrylamide gel at -611 SNP. M marker 20bp, 1 genotype AA, 2 genotype GG, 3 genotype AG. Right) The digestion pattern of Afe1 restriction enzyme on 2% agarose gel at -56 SNP. M marker 50bp, 1,2 genotype TC, 3 genotype TT, 4 genotype CC.

**Table 3:** Genotype Distributions of SNPs in Study Participants

Polymorphism	Genotype	CHP, No. (%)	HCS, No. (%)	P value	OR (95% CI)	Adj OR (95% CI)	Total, No.
**56**				0.002			
	TT	74 (37)	42 (20.9)		1.00 (-)	1.00 (-)	116
	TC	86 (43)	103 (51.7)		0.469 (0.292-0.754)	0.486 (0.301-0.783)	190
	CC	40 (20)	55 (27.4)		0.413 (0.237-0.720)	0.421 (0.221-0.783)	95
**611**				0.006			
	AA	82 (41)	76 (37.8)		1.00 (-)	1.00 (-)	158
	AG	115 (57.5)	117 (53.7)		0.987 (0.656-1.484)	1.00 (0.666-1.512)	223
	GG	3 (1.5)	17 (8.5)		0.164 (0.046-0.580)	0.177 (0.050-0.632)	20

Abbreviations: CHP, chronic hepatitis B patients; HCS, healthy controls; OR, odd’s ratio.

## Discussion

Chronic HBV infection is a multifactorial disease and endemic in Asia and Africa that partialy occurs in Caucasian population. Several immunoregulatory genes and proinflammatory cytokines involved in the host immune response to HBV infection (9, 16), display significant differences among ethnic populations (11). Thus, it was postulated that susceptibility to chronic HBV infection might originate from the genetic variations of host immunity against the virus in different ethnic populations. In this study, 400 Iranian adults were recruited, including 200 chronic hepatitis B patients, and 200 healthy blood donors. Two polymorphisms of IFN-γ R1promoter were identified, including two SNPs at position -56 and -611. Analysis of IFN-γ R1promoter alleles and genotypes distributions in two Iranian groups revealed a significant association between IFN-γ R1promoter polymorphisms and the susceptibility to chronic HBV infection. The results of the current study suggest that carriers of mutant allele T at position -56 are more susceptible to chronic HBV infection and carriers of wild-type allele G at position -611 are predisposed to recover from HBV infection. There is accumulating evidence that the promoter IFN-γ R1polymorphisms have noteworthy association with different chronic infections. Kardom et al. (10) have detected two SNPs positioned at the promoter region of IFN-γ R1in tuberculosis patients. The results of their study showed that the -56 T/C SNP is associated with chronic infection. In a parallel study the polymorphisms within the IFN-γ R1 promoter in company with their promoter functions in a Chinese HBV infected population have been studied. The results of the study suggest that the mutant allele T is a functional SNP and is associated with susceptibility to chronic HBV infection (similar to the current study population at -56 T/C SNP) but no polymorphism have been found at position -611 (19). Genetic polymorphism in the IFN-γ R1gene promoter was linked to atopic cataracts (13). The genetic variations within promoter of IFN-γ R1gene among different ethnics and various cell lines have been studied, a deletion/insertion polymorphism at promoter locus -470, found in Africans , is absent in Europeans and Asians (19). The functional effects of –56C/T SNP have also been investigated. The presence of C allele exhibited a decreased transcriptional activity in reporter gene expression in the human B-cell line and myeloid cell lines whereas at -56T/C SNP mutant allele T in the hHepG2 cell line illustrated a reduction in transcription and wild allele C showed a high level of transcription (15).

It is plausible that chronic HBV infection is a multifactorial disease. The host immunological factors that can be effective in viral persistence are not well understood. Rapid advances in human genomics offer great opportunities to associate genetic variations with risks of diseases. In conclusion, the existence of two polymorphisms within of the IFN-γ R1gene promoter in an Iranian population were detected. According to the results of the current study, especially at position -56 SNP, it seems that allele T can be -considered as a risk factor towards susceptibility to chronic infection in the population under study; and genetic variations in promoter of IFN-γ R1gene may affect the clinical course of HBV Infection.
